# Supplementary material for: Pyruvate Dehydrogenase Kinase 1 inhibition mediated oxidative phosphorylation enhancement in cartilage promotes osteoarthritis progression
Source: BMC Musculoskelet Disord. 2023 Jul 20;24:597. doi: 10.1186/s12891-023-06585-6 (PMC10357736; doi:10.1186/s12891-023-06585-6)
Supplement: Supplementary file 1 — Additional file 1. [file 12891_2023_6585_MOESM1_ESM.zip › MF.Gsea.1653627316242/GOMF_CATALYTIC_ACTIVITY_ACTING_ON_A_GLYCOPROTEIN.html]

Details for gene set GOMF\_CATALYTIC\_ACTIVITY\_ACTING\_ON\_A\_GLYCOPROTEIN[GSEA]

|  || Dataset | 55457\_collapsed\_to\_symbols.cls.cls #OA\_versus\_normal.cls.cls #OA\_versus\_normal\_repos |
| Phenotype | cls.cls#OA\_versus\_normal\_repos |
| Upregulated in class | OA |
| GeneSet | GOMF\_CATALYTIC\_ACTIVITY\_ACTING\_ON\_A\_GLYCOPROTEIN |
| Enrichment Score (ES) | 0.68807405 |
| Normalized Enrichment Score (NES) | 2.1335545 |
| Nominal p-value | 0.0 |
| FDR q-value | 0.0023045691 |
| FWER p-Value | 0.018 |
Table: GSEA Results Summary

  

Fig 1: Enrichment plot: GOMF\_CATALYTIC\_ACTIVITY\_ACTING\_ON\_A\_GLYCOPROTEIN      
 Profile of the Running ES Score & Positions of GeneSet Members on the Rank Ordered List

  

| SYMBOL | TITLE | RANK IN GENE LIST | RANK METRIC SCORE | RUNNING ES | CORE ENRICHMENT || 1 | C1GALT1C1 | C1GALT1 specific chaperone 1 [Source:HGNC Symbol;Acc:HGNC:24338] | 22 | 1.098 | 0.1825 | Yes |
| 2 | MGAT4C | MGAT4 family member C [Source:HGNC Symbol;Acc:HGNC:30871] | 234 | 0.796 | 0.2996 | Yes |
| 3 | MGAT4A | "alpha-1,3-mannosyl-glycoprotein 4-beta-N-acetylglucosaminyltransferase A [Source:HGNC Symbol;Acc:HGNC:7047]" | 657 | 0.604 | 0.3681 | Yes |
| 4 | C1GALT1 | "core 1 synthase, glycoprotein-N-acetylgalactosamine 3-beta-galactosyltransferase 1 [Source:HGNC Symbol;Acc:HGNC:24337]" | 1110 | 0.494 | 0.4159 | Yes |
| 5 | B4GALT7 | "beta-1,4-galactosyltransferase 7 [Source:HGNC Symbol;Acc:HGNC:930]" | 1411 | 0.442 | 0.4667 | Yes |
| 6 | ABO | "ABO, alpha 1-3-N-acetylgalactosaminyltransferase and alpha 1-3-galactosyltransferase [Source:HGNC Symbol;Acc:HGNC:79]" | 1495 | 0.429 | 0.5323 | Yes |
| 7 | FUT8 | fucosyltransferase 8 [Source:HGNC Symbol;Acc:HGNC:4019] | 1565 | 0.418 | 0.5971 | Yes |
| 8 | GCNT3 | "glucosaminyl (N-acetyl) transferase 3, mucin type [Source:HGNC Symbol;Acc:HGNC:4205]" | 1683 | 0.401 | 0.6553 | Yes |
| 9 | GCNT1 | glucosaminyl (N-acetyl) transferase 1 [Source:HGNC Symbol;Acc:HGNC:4203] | 2035 | 0.357 | 0.6881 | Yes |
| 10 | POMGNT1 | "protein O-linked mannose N-acetylglucosaminyltransferase 1 (beta 1,2-) [Source:HGNC Symbol;Acc:HGNC:19139]" | 3641 | 0.191 | 0.5955 | No |
| 11 | GCNT4 | glucosaminyl (N-acetyl) transferase 4 [Source:HGNC Symbol;Acc:HGNC:17973] | 3858 | 0.169 | 0.6071 | No |
| 12 | MGAT5 | "alpha-1,6-mannosylglycoprotein 6-beta-N-acetylglucosaminyltransferase [Source:HGNC Symbol;Acc:HGNC:7049]" | 5086 | 0.066 | 0.5229 | No |
| 13 | MGAT2 | "alpha-1,6-mannosyl-glycoprotein 2-beta-N-acetylglucosaminyltransferase [Source:HGNC Symbol;Acc:HGNC:7045]" | 5317 | 0.046 | 0.5128 | No |
| 14 | MGAT1 | "alpha-1,3-mannosyl-glycoprotein 2-beta-N-acetylglucosaminyltransferase [Source:HGNC Symbol;Acc:HGNC:7044]" | 5407 | 0.040 | 0.5125 | No |
| 15 | MGAT4B | "alpha-1,3-mannosyl-glycoprotein 4-beta-N-acetylglucosaminyltransferase B [Source:HGNC Symbol;Acc:HGNC:7048]" | 5956 | -0.002 | 0.4703 | No |
| 16 | B3GNT3 | "UDP-GlcNAc:betaGal beta-1,3-N-acetylglucosaminyltransferase 3 [Source:HGNC Symbol;Acc:HGNC:13528]" | 8361 | -0.183 | 0.3144 | No |
| 17 | MGAT3 | "beta-1,4-mannosyl-glycoprotein 4-beta-N-acetylglucosaminyltransferase [Source:HGNC Symbol;Acc:HGNC:7046]" | 8920 | -0.226 | 0.3089 | No |
Table: GSEA details [plain text format]

  

Fig 2: GOMF\_CATALYTIC\_ACTIVITY\_ACTING\_ON\_A\_GLYCOPROTEIN      
 Blue-Pink O' Gram in the Space of the Analyzed GeneSet

  

Fig 3: GOMF\_CATALYTIC\_ACTIVITY\_ACTING\_ON\_A\_GLYCOPROTEIN: Random ES distribution      
 Gene set null distribution of ES for **GOMF\_CATALYTIC\_ACTIVITY\_ACTING\_ON\_A\_GLYCOPROTEIN**
